# Targeting the Sigma-1 Receptor via Pridopidine Ameliorates Central Features of ALS Pathology in a SOD1^G93A^ Model

**DOI:** 10.1038/s41419-019-1451-2

**Published:** 2019-03-01

**Authors:** Ariel Ionescu, Tal Gradus, Topaz Altman, Roy Maimon, Noi Saraf Avraham, Michal Geva, Michael Hayden, Eran Perlson

**Affiliations:** 1 Department of Physiology and Pharmacology, Sackler Faculty of Medicine, Tel Aviv University, Tel Aviv, 69978 Israel; 2 Sagol School of Neuroscience, Tel Aviv University, Tel Aviv, 69978 Israel; 3 Teva Pharmaceuticals Ltd, Petah Tikva, Israel; 4 Prilenia Therapeutics, Herzliya, Israel

## Abstract

Amyotrophic Lateral Sclerosis (ALS) is a fatal neurodegenerative disease affecting both the upper and lower motor neurons (MNs), with no effective treatment currently available. Early pathological events in ALS include perturbations in axonal transport (AT), formation of toxic protein aggregates and Neuromuscular Junction (NMJ) disruption, which all lead to axonal degeneration and motor neuron death. Pridopidine is a small molecule that has been clinically developed for Huntington disease. Here we tested the efficacy of pridopidine for ALS using in vitro and in vivo models. Pridopidine beneficially modulates AT deficits and diminishes NMJ disruption, as well as motor neuron death in SOD1^G93A^ MNs and in neuromuscular co-cultures. Furthermore, we demonstrate that pridopidine activates the ERK pathway and mediates its beneficial effects through the sigma-1 receptor (S1R). Strikingly, in vivo evaluation of pridopidine in SOD1^G93A^ mice reveals a profound reduction in mutant SOD1 aggregation in the spinal cord, and attenuation of NMJ disruption, as well as subsequent muscle wasting. Taken together, we demonstrate for the first time that pridopidine improves several cellular and histological hallmark pathologies of ALS through the S1R.

## Introduction

ALS is a progressive, MN disease manifested by loss of control over voluntary muscles, which leads to death between 3–5 years after diagnosis. Currently, there is no effective treatment for ALS. Familial and sporadic forms of ALS share several key cellular pathologies, including NMJ disruption^1,2^, defects in AT^3^, formation of toxic aggregates^4^ and loss of MNs in the spinal cord and cortex.

The most studied mutation of familial ALS is in the Cu/Zn Superoxide Dismutase-1 (*SOD1*) gene^5^. Transgenic mice expressing the mutated human SOD1 (SOD1^G93A^) gene exhibit pathologies similar to ALS in human patients^6^.

Although the mechanism underlying the disease is still not fully understood, it is thought that loss of NMJs and MNs in ALS may be related to defects in AT. AT is required for the efficient supply and clearance of cellular organelles and proteins essential to axons and NMJs, as well as for regulating vital spatiotemporal signaling events with high specificity and fidelity. Evidently, mutations in AT machinery can cause ALS and motor deficits^7,8^.

Pridopidine is a novel compound that has been under development for the treatment of Huntington disease (HD). Accumulating evidence has suggested that pridopidine mediates its beneficial effects through high-affinity binding to S1R at pharmacologically applicable doses^9–11^. Pridopidine can upregulate the BDNF pathway in neurons via a S1R-dependent mechanism, leading to increased BDNF secretion^11^.

S1R functions as a molecular chaperone in the mitochondria-associated ER membrane (MAM), where it obtains high potency in protein folding^12,13^, and it also regulates ER-mitochondria Ca^2+^ signaling and cell survival^14^. Moreover, the S1R was shown to suppress the generation of ROS^15^. Interestingly, tissue expression profile of S1R in the nervous system reveals that it is mostly expressed in the brainstem and in spinal MNs^16,17^. Indeed, deletion of S1R in mice leads to motor defects^18,19^, and a deletion of S1R in SOD1^G93A^ mice, exacerbates disease progression^20^. Importantly, mutations in the S1R gene cause juvenile and adult forms of ALS^21–23^.

Here we have tested the efficacy of pridopidine in improving fundamental ALS-linked pathologies in SOD1^G93A^ mutants.

## Results

### Pridopidine restores NMJ activity in a neuromuscular co-culture model of ALS

**Fig. 1:**
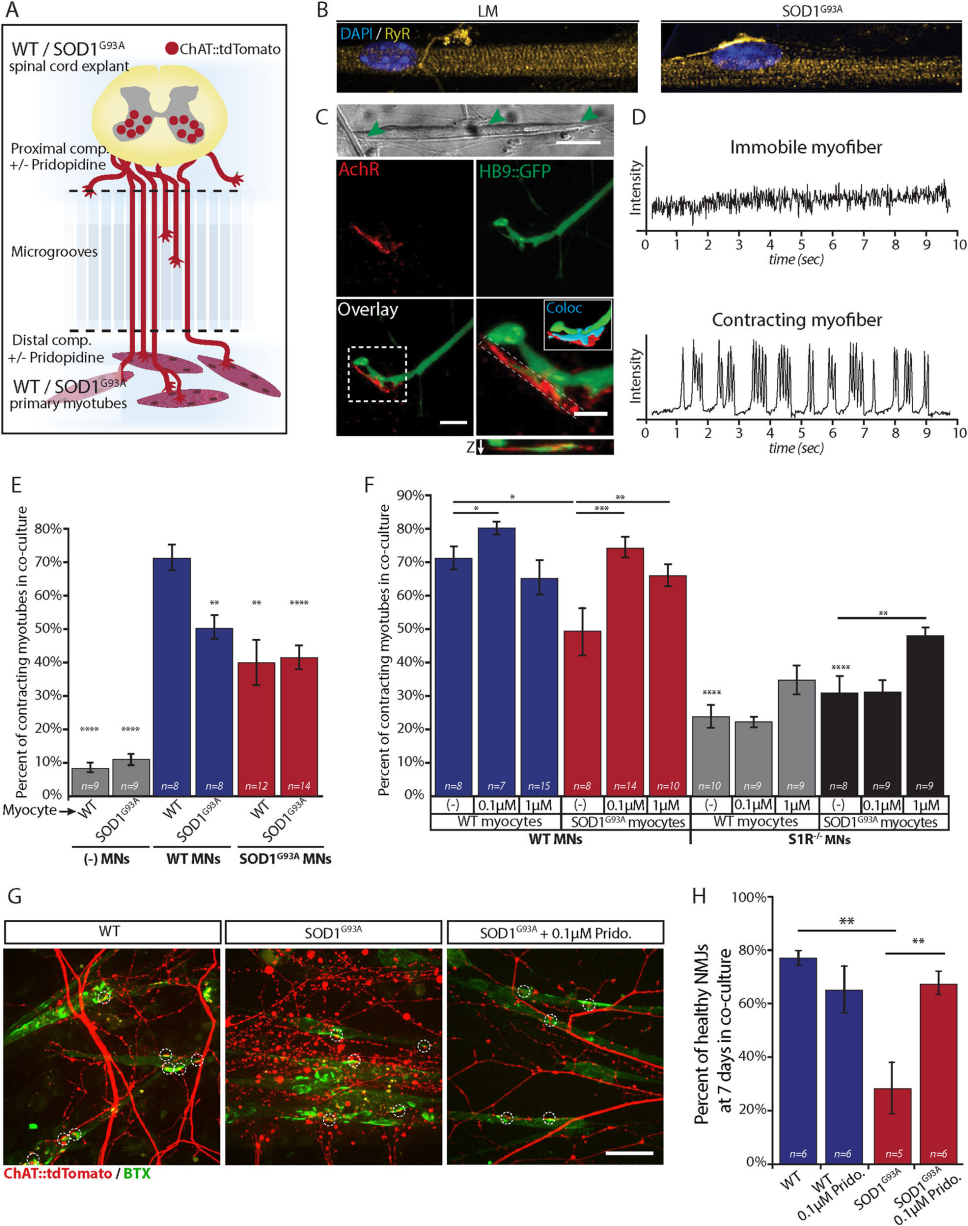
Pridopidine limits the disruption of NMJs in SOD1^G93A^ and restores synaptic activity in SOD1^G93A^ muscle: WT MN co-culture. **a** Schematic illustration of the experimental procedure. Primary skeletal myoblasts from presymptomatic (P60) WT or SOD1^G93A^ mice are cultured in the distal compartment of a microfluidic chamber. Seven days later, ventral spinal cord explants from WT or SOD1^G93A^ E12.5 mouse embryos are plated in the proximal compartment. After 7 more days, axons extend into the distal compartment and generate connections and NMJs with differentiated myocytes. **b** High-magnification fluorescent images of differentiated myotubes in the NMJ compartment labeled for nuclei (blue) and the Ryanodine Receptors (RyR, yellow). Images demonstrate that WT (left) and SOD1^G93A^ (right) myocytes differentiate to the same extent, as can be seen by the peripheral localization of the nuclei, and by the striated organization of RyR. **c** Upper panel: Phase image of a myocyte in the distal compartment connected by axons (green arrowheads). Scale bar: 20 µm. Lower panel: High magnification images of myocyte:MN contact points reveals the formation of NMJs as seen by co-localization of post-synaptic AChRs (red) with HB9::GFP axons (green) and 3-dimensional colocalization of pre- and post-synaptic markers (Cyan). The image below the inset shows an orthogonal slice displaying Z-axis colocalization. Scale bars: 10 µm; inset 5 µm. **d** Muscle contraction traces as extracted from intensity over time measurements of muscle contraction show the flat trace of a non-contracting, immobile myocyte (upper), and the trace of a contracting myocyte demonstrating multiple bursting events (lower). **e** Bar chart of the percentage of contracting myofibers in co-culture shows that only ~10% of muscles contract in the absence of MNs versus 74% in healthy co-cultures. Co-culture combinations that include at least one of the cell types expressing SOD1 show a significantly lower percentage of contracting myocytes. (n = number of microfluidic chambers from 3 independent experiments). **f** Bar chart of the percentage of contracting myocytes in co-culture show that the addition of pridopidine to the culture medium of co-cultures with SOD1^G93A^ myocytes (0.1 and 1 µM) restores neuromuscular activity to the WT levels. Combination of S1R^−/−^ MNs results in even a lower number of contracting myotubes. Application of 0.1 µM pridopidine to S1R^−^^/^^−^ co-cultures does not restore the neuromuscular activity, as seen for the same concentration of pridopidine in co-cultures with WT neurons. Application of 1 µM pridopidine leads to an increase in the percentage of contracting myocytes that is significantly lower than untreated and treated WT cultures. **g** Representative images of *in-vitro* NMJs in the distal compartment display co-localized presynaptic motor axons (red) and AchR patches (green) on myocytes (circled by white dashed lines). The left panel shows that the co-localized axons in the WT co-cultures are intact, whereas axons in SOD1^G93A^ co-cultures are degenerated (center panel). SOD1^G93A^ co-cultures treated with 0.1 µM pridopidine display healthy NMJs and a reduced number of degenerated axons (right panel). Scale bar: 40 µm. **h** Quantitative analysis of in vitro NMJs reveals a profound reduction in the percentage of healthy NMJs in SOD1^G93A^ co-cultures that is significantly moderated by 0.1 µM pridopidine treatment. Data are shown as mean ± SEM. *p value < 0.05; **p value < 0.01, ***p value < 0.001, ****p value < 0.0001. (*n* = number of microfluidic chambers from 3 or more independent experiments; Student’s *t* test)

In order to study the possible beneficial effects of pridopidine on ALS pathology, we used our unique simplified motor unit system^24,25^, co-culturing embryonic ventral spinal cord explants (vSCE) and primary myocytes in compartmental microfluidic chambers (MFCs). This simplified platform allows precise control, monitoring and manipulation of subcellular microenvironments that may impact MN survival, function, and NMJ integrity (Fig. 1a). We demonstrated that primary myocytes within this system differentiate and form connections with MN axons, and that these connections are positive for pre- and post-synaptic markers (Fig. 1b, c). Furthermore, we also developed an image-based method sensitive enough to detect functional NMJs by analyzing the contractile behavior of innervated myocytes^24^ (Fig. 1d). Since NMJ disruption may be an early event in ALS patients^2^, we sought first to determine the effects of only diseased MNs or diseased myocytes on NMJ activity. Testing the percentage of contracting muscles in the absence of MNs demonstrated that healthy (WT) and SOD1^G93A^ denervated myocytes hardly contract (Fig. 1e). Next, we co-cultured combinations of WT or SOD1^G93A^ MNs with WT or SOD1^G93A^ myocytes, and compared the number of contracting innervated-myocytes. The presence of diseased MNs results in a significant reduction in active NMJs in these co-cultures. Strikingly, SOD1^G93A^ myocytes alone are sufficient to generate NMJ dysfunction when cultured with healthy neurons, suggesting a role for muscles in the disease’s etiology.

Next, we tested the outcome of applying pridopidine to these co-cultures. The presence of pridopidine restored NMJ activity in diseased co-cultures, and it also increased NMJ activity in WT cultures even further.

Following that, we visualized the state of NMJ health in WT and SOD1^G93A^ co-cultures. To this end, we used SCE from a MN reporter mouse that expresses tdTomato under the ChAT promotor (ChAT::tdTomato) (Fig. 1a). By crossing these mice with SOD1^G93A^ mice, we were able to isolate SCE from WT^ChAT::tdTomato^ and SOD1^G93A/ChAT::tdTomato^ embryos and co-culture them with WT and SOD1^G93A^ myocytes, respectively. Co-cultures were fixed after 7 days, and stained with α-bungarotoxin-FITC (BTX) to visualize AchRs. High-magnification images of the NMJ compartment reveal massive axon degeneration and NMJ disruption in the SOD1^G93A^ co-cultures, whereas ~ 75% of NMJs remain intact in WT co-cultures (Fig. 1g).

Quantifying the percentage of healthy NMJs reveals a strikingly improved condition in SOD1^G93A^ co-cultures treated with pridopidine (0.1 µM), suggesting that low dose of pridopidine in these co-cultures is sufficient to retrieve the WT phenotypes, whereas higher dosage (1 µM) may result in conflicting outcomes. Thus, our in vitro experiments suggest that pridopidine prevents NMJ disruption and restores their activity in SOD1^G93A^ cultures.

### Pridopidine impedes MN death in co-cultures of SOD1^G93A^

**Fig. 2:**
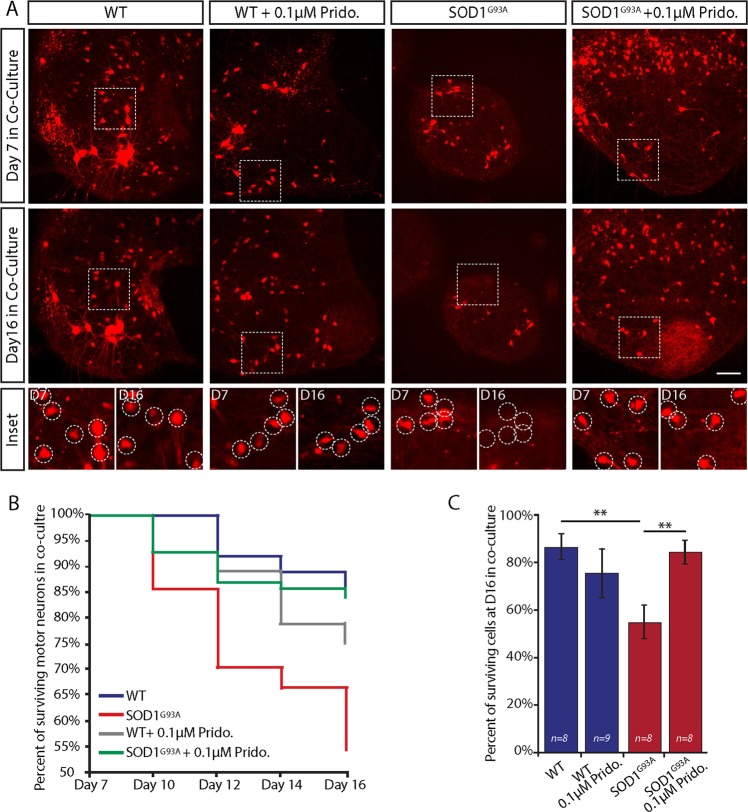
Pridopidine impedes motor neuron loss in co-cultures of SOD1^G93A^. **a** Representative images of either pridopidine treated or control SOD1^G93A/ChAT::tdTomato^ and WT^ChAT::tdTomato^ spinal cord explants in the proximal compartment of MFCs in co-culture. Upper panel shows the ChAT-positive motor neurons (red) within each spinal cord explant at day 7 of co-culture. Central panel shows images of the same spinal cords at day 16 of co-culture. Bottom panel includes magnified images of the regions from the upper panels denoted by a dashed line. Images display the massive death of motor neurons in the SOD1^G93A^ explants in co-cultures, whereas the number of surviving motor neurons in the WT and pridopidine-treated SOD1^G93A^ remains roughly the same. Dashed circles denote motor neurons. **b** Plot of the average percentage of surviving motor neurons per explant over time displays accelerated motor neuron death in the SOD1^G93A^ co-cultures that is profoundly impeded by 0.1 µM pridopidine application. **c** Bar chart showing the percentage of surviving cells per explant at day 16 in co-culture, which reveals a substantial rescue effect for pridopidine on SOD1^G93A^ motor neurons. Data are shown as mean ± SEM. **p value < 0.01. (*n* = number of spinal cord explants from 3 or more independent experiments; Student’s *t* test.)

To study if pridopidine can alleviate MN death, we tracked the number of MNs within the SCE of WT^ChAT::tdTomato^ and SOD1^G93A/ChAT::tdTomato^ in co-culture over time (Fig. 2). Explants were monitored at 7, 10, 12, 14, and 16 days of co-culture. Our observations indicate a massive neuronal death in the SOD1^G93A^ co-cultures which began at day 10 and progressed rapidly up to ~50% loss of MNs at day 16 (Fig. 2a, b). Evidently, pridopidine (0.1 µM) markedly delays the death of MNs in the SOD1^G93A^ co-cultures (Fig. 2c).

Noteworthy, our results indicate that the massive death of MNs in SOD1^G93A^ begins at day 10, shortly after NMJ disruption and axonal degeneration, therefore supporting a dying-back mechanism for ALS^2^.

The evident potency of pridopidine to ameliorate the cellular phenotypes of ALS led us to investigate the mechanism by which pridopidine facilitates its beneficial effects.

### Pridopidine enhances AT in MNs and repairs the AT defects in SOD1^G93A^ MNs

**Fig. 3:**
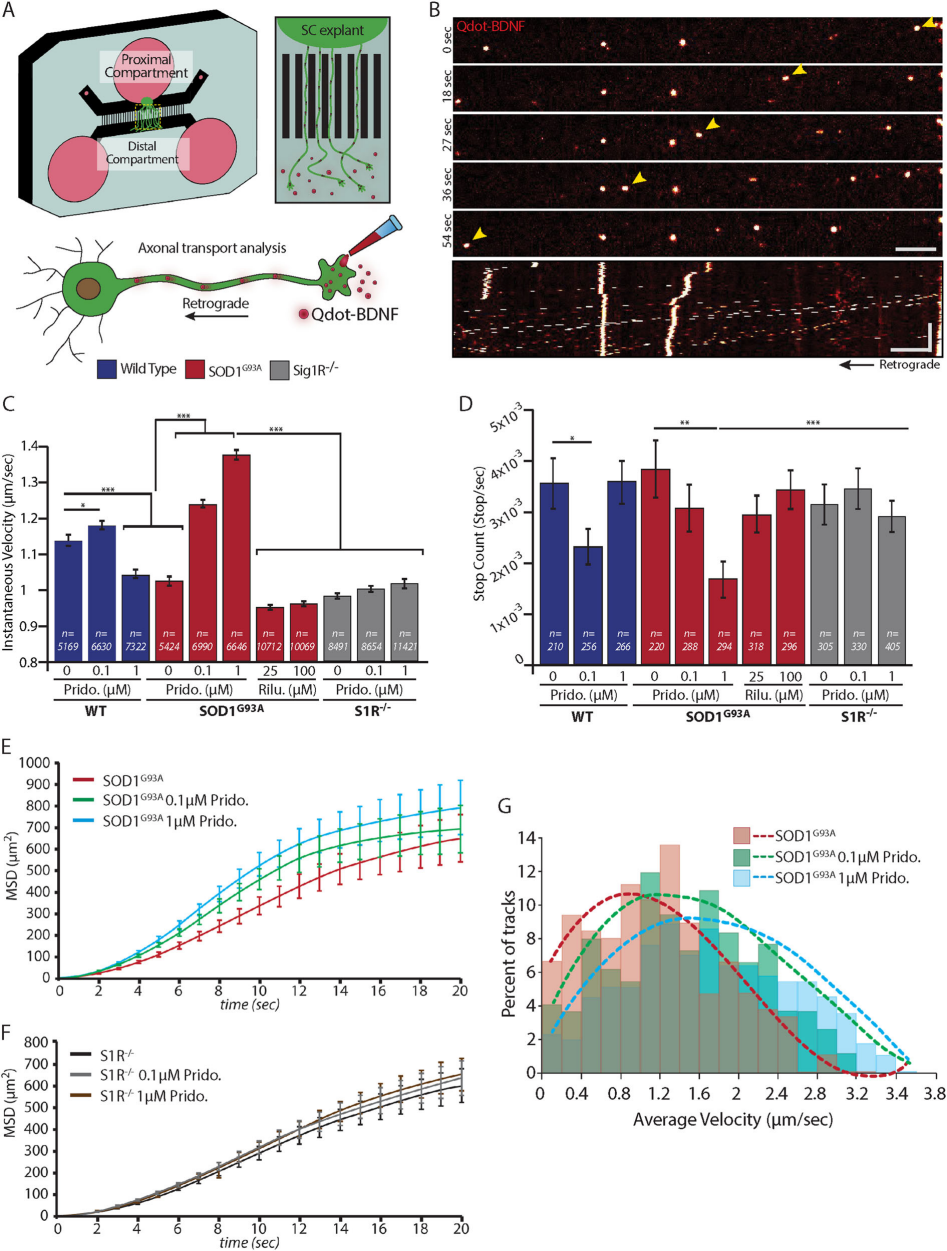
Pridopidine repairs axonal transport defects in SOD1^G93A^ MNs and further enhances axonal transport to a processive mode. **a** Schematic illustration of an experimental system for axonal transport tracking in MNs. Spinal cord (SC) explants from WT or SOD1^G93A^ E12.5 mouse embryos are plated in the proximal compartment of a microfluidic chamber. MNs from the spinal cord send their axons towards the distal compartment. At this point Qdot-BDNF is applied exclusively to this compartment. Axonal transport of Qdot-BDNF is then visualized and recorded by a high-resolution spinning disk confocal imaging system. **b** Time lapse images of Qdot-BDNF (bright orange) axonal transport as acquired at 60X magnification. Yellow arrowheads point to a single Qdot-BDNF particle that is retrogradely transported (left) towards the cell body. Scale bar: 10 µm. Bottom panel shows a kymograph of a complete Qdot-BDNF time-lapse movie. Scale bars: Horizontal 10 µm; vertical 100 s. **c** Bar chart of the Instantaneous Velocity values for Qdot-BDNF particles in WT (blue) or SOD1^G93A^ (red) MNs show slower velocities in the SOD1^G93A^ MNs. Pridopidine application accelerates the instantaneous velocities both in WT MNs (0.1 µM) and SOD1^G93A^ MNs (0.1 µM and 1 µM). Application of 25 µM or 100 µM Riluzole on SOD1^G93A^ MNs does not affect the instantaneous velocities. S1R^−^^/^^−^ MNs reveal defects in the axonal transport of BDNF. Pridopidine at either 0.1 µM or 1 µM was unable to recover these defects (n = number of qDot-BDNF steps). **d** Bar chart of particle Stop-Count shows that pridopidine reduces the number of pauses during axonal transport in WT MNs (0.1 µM only) and SOD1^G93A^ MNs (both 0.1 µM and 1 µM). Axonal transport parameters of Qdot-BDNF in S1R^−^^/^^−^ MNs show that unlike SOD^G93A^ MNs, they are not responsive to pridopidine at any of the concentrations tested (n = number of Qdot-BDNF tracks). **e**, **f** Graphs showing the MSD over time for SOD1^G93A^ MNs **e** and S1R^−^^/^^−^ MNs **f** show that pridopidine accelerates axonal transport in a S1R-dependent mechanism. **g** Distribution of average velocities of analyzed tracks in SOD1^G93A^ MNs shows a significant increase towards higher velocities in pridopidine-treated MNs. Dashed lines denote a polynomial fitting curve for each condition. Data are shown as the mean ± SEM. *p value < 0.05; **p value < 0.01; ***p value < 0.001 (*n* = 6 independent experiments; the sample size for each experiment is indicated on bars; Student’s *t* test)

Defects in AT have been previously described in models of ALS^26^, as well as in several other neurodegenerative diseases^27^. In order to visualize and analyze AT, we plated SCE from WT or SOD1^G93A^ embryos in the proximal compartment of an MFC (Fig. 3a). We tracked the retrograde AT of Qdot-BDNF particles that were introduced exclusively to axons in the distal compartment using live imaging (Fig. 3b).

Our analysis revealed a significant reduction in the instantaneous velocity (*V*_*inst*_*)* of Qdot-BDNF in SOD1^G93A^ MN (Fig. 3c). Remarkably, overnight treatment of cultures with 0.1 or 1 µM pridopidine accelerated the *V*_*inst*_ in SOD1^G93A^ MN in a concentration-dependent manner, whereas the effect on WT neurons is milder and exhibited a concentration-independent behavior. Notably, overnight treatment with 25 and 100 µM of Riluzole^28^, the current medication for ALS, did not have a similar effect. The average number of stops during particle transport was similar in untreated WT and SOD1^G93A^ MN (Fig. 3d). Nevertheless, pridopidine reduced the stop-count in SOD1^G93A^ to a greater extent than in WT neurons, which might explain the overcompensation observed in *V*_inst_. Further analysis of the Mean Square Displacement (MSD) in SOD1^G93A^ revealed a significant elevation in the displacement of Qdot-BDNF in pridopidine-treated MNs (Fig. 3e, f). The increase in *V*_inst_, and the reduction in the stop-count eventually translate to a significant increase in the average velocity (*V*_avg_) of particles in SOD1^G93A^ MNs (Fig. 3g). Taken together, these results indicate that pridopidine treatment improves AT defects in SOD1^G93A^ MNs and further accelerates and shifts the AT to a more directed mode.

### Pridopidine beneficially modulates AT in SOD1^G93A^ neuromuscular co-cultures

**Fig. 4:**
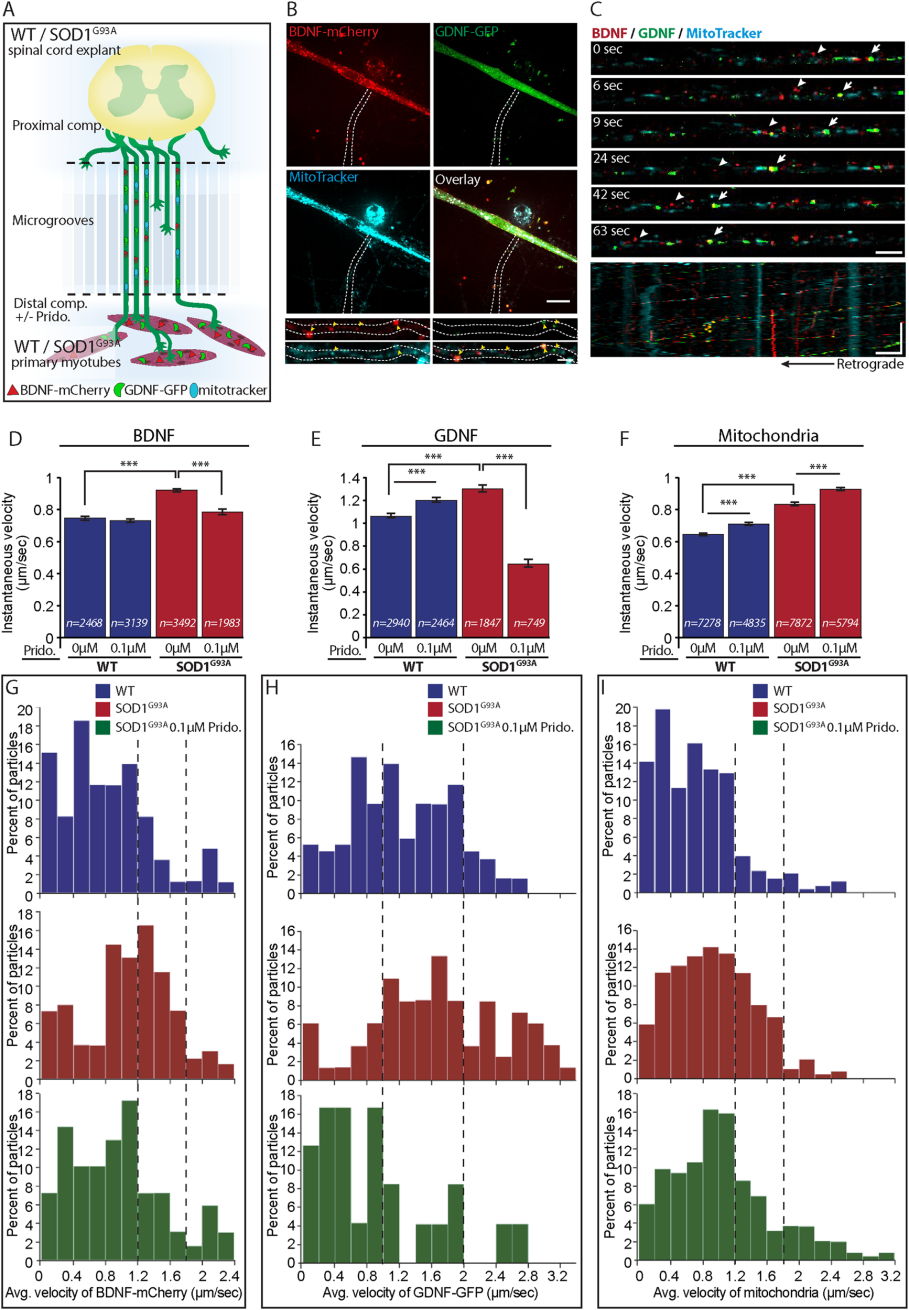
Locally administered Pridopidine regulates the axonal transport of BDNF, GDNF, and mitochondria in co-culture of SOD1^G93A^. **a** Schematic illustration of the experimental procedure. Primary skeletal myoblasts expressing BDNF-mCherry and GDNF-GFP are co-cultured with ventral spinal cord explants from WT or SOD1^G93A^ E12.5 mouse embryos. Pridopidine is exclusively applied at this phase to the distal compartment. At 7 days in co-culture, the axonal transport of muscle-derived BDNF-mCherry and GDNF-GFP, as well as the transport of mitochondria is imaged. **b** Representative confocal images of myocyte in the distal compartment expressing both BDNF-mCherry (red) and GDNF-GFP (green) and stained with MitoTracker (cyan). The white dashed line denotes an axon innervating a muscle containing BDNF-mCherry and GDNF-GFP particles as well as mitochondria. Scale bar: 20 µm. Lower panel includes magnified images of the axon. Yellow arrowheads denote the BDNF, GDNF, and mitochondria within the axon. Scale bar: 5 µm. **c** Representative time-lapse images show the retrograde axonal transport of muscle-derived BDNF-mCheery (red), GDNF-GFP (green), as well as the retrograde transport of mitochondria (cyan). White arrows denote co-transported BDNF-mCherry and GDNF-GFP. The arrowhead denotes co-transported BDNF-mCherry and mitochondria. Scale bar: 5 µm. Lower panel includes a kymograph of the entire time lapse movie demonstrating that most of the BDNF and GDNF, as well as mitochondria are retrogradely transported. Scale bars – horizontal: 5 µm vertical: 100 s. **d–f** Quantitative analysis of the instantaneous velocity of D) BDNF-mCherry, E) GDNF-GFP, and F) mitochondria from axons in co-culture of WT or SOD1^G93A^ reveals accelerated axonal transport of all of the above in SOD1^G93A^ compared to WT (*n* = number of particle steps). Application of 0.1 µM pridopidine exclusively to the distal compartment is sufficient to restore the axonal transport properties of BDNF-mCherry and GDNF-GFP. However, the retrograde mitochondrial transport is further accelerated in the pridopidine-treated cultures. **g–i** Stacked distribution plots of the average velocity of G) BDNF-mCherry, H) GDNF-GFP, and I) mitochondria display higher transport velocities for all of the above in SOD1^G93A^ co-cultures. dashed lines highlight the changes in transporting populations of GDNF-GFP, BDNF-mCherry and mitochondria. Whereas 0.1 µM pridopidine moderates the velocities of BDNF-mCherry and GDNF-GFP, a specific population of mitochondria is transported even faster in response to the pridopidine treatment. Data are shown as the mean ± SEM. ***p value < 0.001 (*n* = 3 or more independent experiments; Student’s *t* test)

We next sought to test the AT properties of SOD1^G93A^ in co-culture to better mimic mature motor units (Fig. 4a). Myocytes were transfected with both BDNF-mCherry and GDNF-GFP and differentiated for 7 days in the MFC, after which SCE were plated in the proximal compartment. At 7 days in co-culture, mitochondria were stained with MitoTracker for enabling their tracking in MNs as well, and AT time-lapse movies of BDNF-mCherry, GDNF-GFP and MitoTracker were acquired (Fig. 4b). Our recordings in both WT and SOD1^G93A^ co-cultures reveal that BDNF-mCherry and GDNF-GFP are both secreted from muscles, transported into MNs and move mostly retrogradely within the axons (Fig. 4c). Interestingly, we also identified co-transporting particles, suggesting the existence of various populations of transported cargo that might have different roles, destinations, and transport properties (Fig. 4c).

Extended analysis of the AT in these co-cultures revealed unexpected results. Both the *V*_inst_ (Fig. 4d–f) and *V*_avg_ (Fig. 4g–i) of BDNF-mCherry, GDNF-GFP, and mitochondria were significantly increased in the SOD1^G93A^ co-cultures. Nevertheless, applying 0.1 µM of pridopidine exclusively in the NMJ compartment, was sufficient to decrease the AT of BDNF-mCherry and GDNF-GFP back to WT levels. Notably, in presence of pridopidine in SOD1^G93A^ co-cultures, we could hardly detect GDNF-GFP particles moving retrogradely (data not shown). Intriguingly, the *V*_inst_ of mitochondria in the pridopidine-treated co-cultures was increased even further in both WT and SOD1^G93A^ co-cultures. Distribution plots of mitochondrial *V*_avg_ point to a specific subpopulation of mitochondria moving much faster (Fig. 4i).

### Pridopidine activates ERK but not AKT in WT and SOD1^G93A^ MNs

**Fig. 5:**
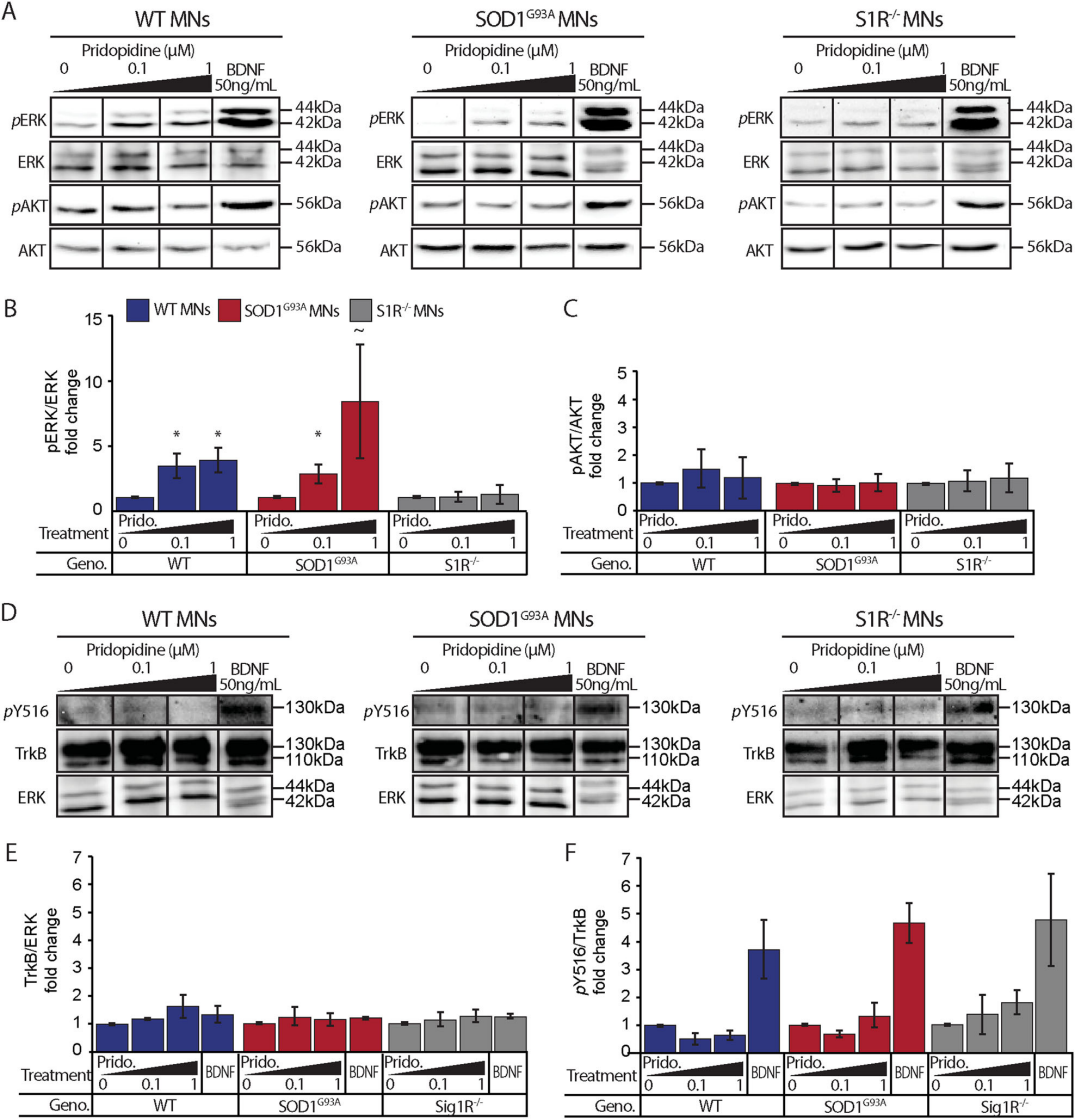
Pridopidine increases ERK but not AKT and trkB phosphorylation in MNs. **a** Western blots of WT, SOD1^G93A^, and S1R^−^^/^^−^ MN culture extracts show an increase in the phosphorylation of ERK following a 30-minute incubation with 0.1 µM and 1 µM pridopidine in WT MNs (left panel) and SOD1^G93A^ (middle panel), but not in S1R^−^^/^^−^ MNs (right panel). AKT phosphorylation remained unaffected by pridopidine. Importantly, 50 ng/mL BDNF stimulation activates ERK and AKT in MNs from all sources. **b** Quantification of ERK reveals a ~3.5-4-fold increase in ERK phosphorylation in WT MNs following 0.1 µM and 1 µM pridopidine, respectively. SOD1^G93A^ exhibited ~2.9-8.5-fold increase in ERK phosphorylation following 0.1 µM and 1 µM pridopidine, respectively. Pridopidine did not lead to ERK phosphorylation in S1R^−^^/^^−^ MNs. **c** Quantification of AKT phosphorylation did not reveal any differences following pridopidine treatment. **d** Western blots of WT, SOD1^G93A^, and S1R^−^^/^^−^ MN culture extracts show that the levels of TrkB and phospho(Thy516)TrkB in WT (left) SOD1^G93A^ (center) and S1R^−^^/^^−^ motor neurons do not change in response to either 0.1 µM or 1 µM pridopidine. BDNF 50 ng/mL was used as a positive control to demonstrate phospho(Thy516)TrkB activation in all MNs from all sources. **e** Quantification of TrkB levels reveals no change in its expression under all conditions. **f** Quantification of TrkB phosphorylation reveals that pridopidine does not activate theTrkB receptor at any of the tested concentrations. Data are shown as the mean pERK/ERK, pAKT/AKT, pTrkB/TrkB, or TrkB/Tubulin ratios ± SEM. *p value < 0.05, ~p value < 0.1 (*n* = 3 independent experiments; Student’s *t* test.)

ERK and AKT are key signaling pathways that regulate many cellular processes, including neurotrophic ones^24,29^. We sought to determine whether pridopidine could activate ERK or AKT pathways in WT and SOD1^G93A^ MNs, and therefore play a neuroprotective role via one of these pathways. Primary MN cultures at 2DIV were starved overnight in neurotrophin- and serum-free medium (PNB). The following day, cultures were treated for 30 min with pridopidine or with BDNF as a positive control. Western-blot analyses for phosphorylated-ERK (pERK) reveal a significant activation of ERK (Fig. 5a, b).

ERK activation in pridopidine-treated SOD1^G93A^ MNs was to the same extent as in WT MNs. Interestingly, we did not detect any change in the phosphorylation level of AKT following application of pridopidine (Fig. 5c). Since both ERK and AKT activation can be downstream to the activation of neurotrophin receptor, TrkB^30^, we investigated whether pridopidine modulates the expression or activation of this receptor. Western-blot analysis suggests that pridopidine does not affect the expression levels, nor the phosphorylation of Y516^31^ residue of TrkB in MNs (Fig. 5d–f).

Next, we investigated whether pridopidine influences BDNF secretion from myocytes. Hence, we transfected WT and SOD1^G93A^ myocytes with BDNF-mCherry. Following 7-day differentiation, pridopidine (0.1 µM) was added for overnight incubation. Western-blot analysis of BDNF-mCherry in conditioned media compared to its total level in the culture extracts, of the SOD1^G93A^ but not the WT, showed a mild increase in the secretion of BDNF-mCherry following pridopidine application (Supplementary Figure 2). Hence, pridopidine could promote neuronal survival and ameliorate NMJ disruption through activation of the ERK pathway via a neurotrophin- and TrkB(pY516)-independent mechanism.

### Pridopidine facilitates its neuroprotective effects through the S1R pathway

Mutations in the S1R gene were recently reported to cause various forms of ALS including a juvenile form^21–23^. Furthermore, reports by several groups suggest that pridopidine mediates its protective effects via the S1R pathway in neurodegenerative models of HD^9,10^. Thus, we sought to determine whether the neuroprotective effects we observed are reproducible in MNs from S1R^−^^/^^−^ mice^18^.

SCE from S1R^−^^/^^−^ embryos were cultured in the MFC as described before, and the contractile behavior of SOD1^G93A^ or WT myocytes was tested. Strikingly, co-cultures with S1R^−^^/^^−^ SCE exhibited a severe disruption of neuromuscular activity when combined with either healthy or diseased myocytes (Fig. 1f). Pridopidine at 0.1 µM was insufficient to restore NMJ activity to the WT levels, suggesting that pridopidine acts via the S1R. Surprisingly, we did observe a minor increase in the percentage of contracting muscles at the 1 µM concentration, suggesting that pridopidine either acts also on muscles, or that higher concentrations may bind low-affinity targets and activate alternative pathway.

Co-culturing S1R^−^^/^^−^ primary myocytes with WT MNs, did not demonstrate a similar disruption of neuromuscular activity as in the S1R^−^^/^^−^ MNs co-cultures (Fig. 1f), and under these conditions pridopidine did not increase the percentage of contracting muscles any further (Supplementary Figure 3). Next, we analyzed the AT of Qdot-BDNF in S1R^−^^/^^−^ MNs. Our observations revealed that the *V*_*inst*_ of Qdot-BDNF is significantly reduced in S1R^−^^/^^−^ MNs, and is reminiscent of the SOD1^G93A^ one (Fig. 3c). Importantly, unlike in WT or SOD1^G93A^ MNs, pridopidine did not facilitate AT in S1R^−^^/^^−^ MNs in any of the tested parameters and concentrations (Fig. 3d–f). Finally, testing for ERK-pathway activation in S1R^−^^/^^−^ MNs revealed that deletion of S1R in MNs hinders the pridopidine-mediated ERK phosphorylation (Fig. 5a, b). Interestingly, encouraged by the hypothesis that S1R levels might be altered in SOD1^G93A^ tissues, and in different compartments of MNs, we carried out western-blot analyses of MN cultures, as well as analyses of brain, spinal-cord, sciatic-nerve, and muscle tissues from these mice. SOD1^G93A^ tissues displayed variable levels of S1R but we could not detect any appreciable differences in its levels (Supplementary Figure 1). Thus, our data suggest that pridopidine restores NMJ activity and facilitates AT in SOD1^G93A^ in a S1R-dependent mechanism.

### Pridopidine sub-cutaneous administration leads to reduced hSOD1 aggregates in the spinal-cords of SOD1^G93A^ mice

**Fig. 6:**
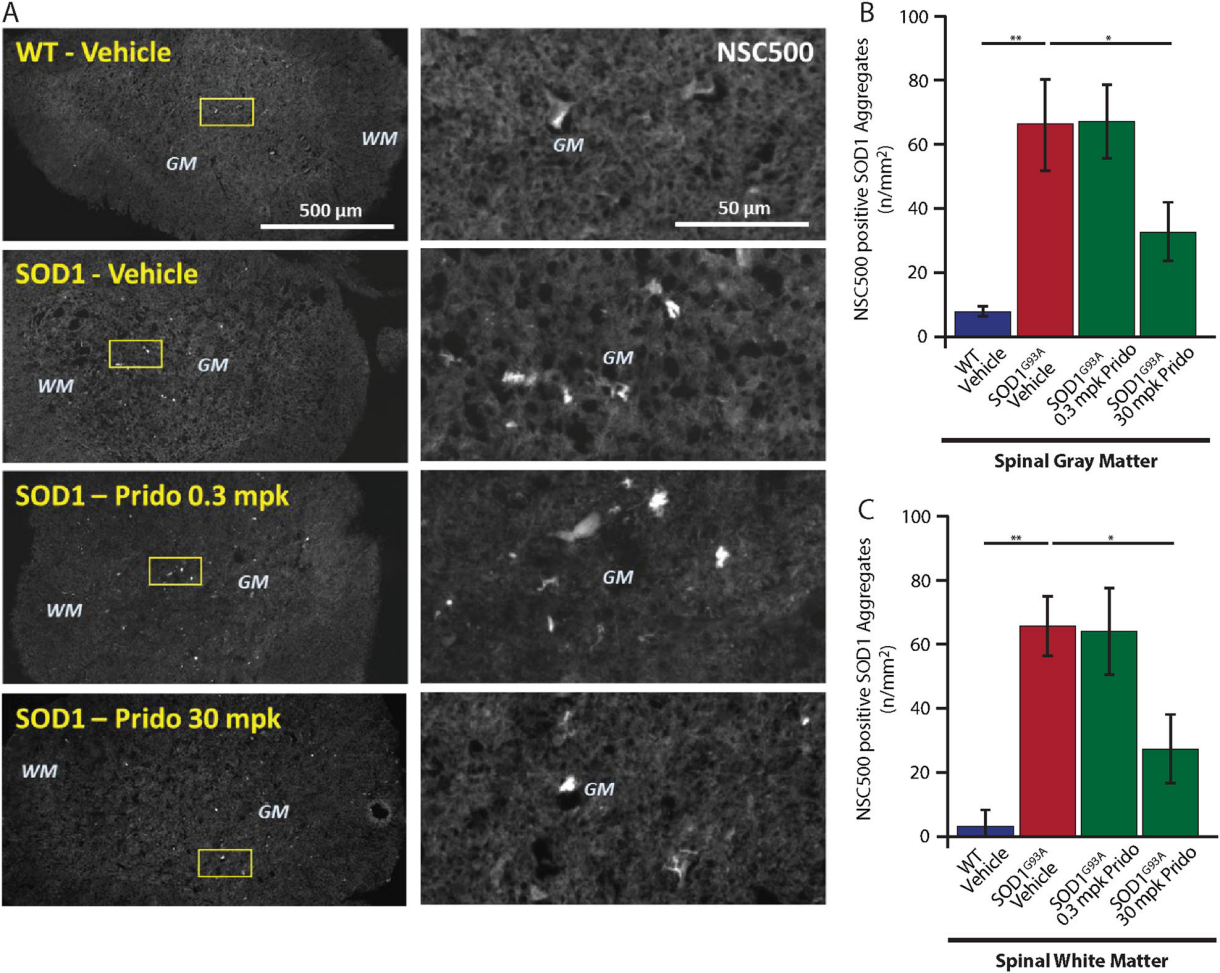
Pridopidine eliminates mSOD1 aggregates in SOD1^G93A^ spinal cords. **a** Left panel: low magnification representative images of fluorescently labeled spinal cords for 4 mouse groups. Top to bottom: WT vehicle, SOD1^G93A^ vehicle, SOD1^G93A^ 0.3 mg/kg (mpk) pridopidine, SOD1^G93A^ 30mpk, all stained with NSC500 to label mSOD1 protein aggregates. Images identify an increase in the amount of aggregates in SOD1^G93A^ compared to almost none in WT controls. Spinal cords from 30mpk, but not 0.3mpk pridopidine-treated mice show a notable decrease in the number of aggregates. Right panel: high-magnification images for the regions marked in the left panel by a square. Scale bars: Left panel: 500 µm; Right panel 50 µm. **b**, **c** Quantitative analysis of the number of aggregates per area identified a significant increase in the number of mSOD1 aggregates in both the gray and white matter of the spinal cords of SOD1^G93A^ mice compared with WT mice. Pridopidine at 30mpk significantly reduces the number of aggregates in the gray (B) and white (C) matter of SOD1^G93A^ spinal cords by ~50%. Data are shown as the mean ± SEM. *p value < 0.05; **p value < 0.01 (*n* = 4 mice in each group; one-way ANOVA followed by Fisher’s LSD post hoc tests.)

S1R functions as a molecular chaperone partly localized to the MAM^12^, and its deletion in mice increases protein aggregation^13^. Thus, we sought to determine the in vivo effect of pridopidine administration on mSOD1 aggregation. SOD1^G93A^ and WT mice were subcutaneously (SC) administered with pridopidine daily for 11-weeks at 3 mg/kg or 30 mg/kg. Control mice were treated with saline. As expected, by staining spinal-cord sections with NSC500 to label mSOD1 aggregates^32^, we identified a significantly higher number of SOD1 aggregates in the gray, as well as the white matter of spinal-cords from SOD1^G93A^ vehicle-treated mice (Fig. 6a–c). Strikingly, daily SC administration of pridopidine at 30 mg/kg eliminated ~50% of SOD1 aggregates in the spinal-cords of SOD1^G93A^ mice.

### Pridopidine ameliorates muscle fiber atrophy and preserves NMJs in vivo

**Fig. 7:**
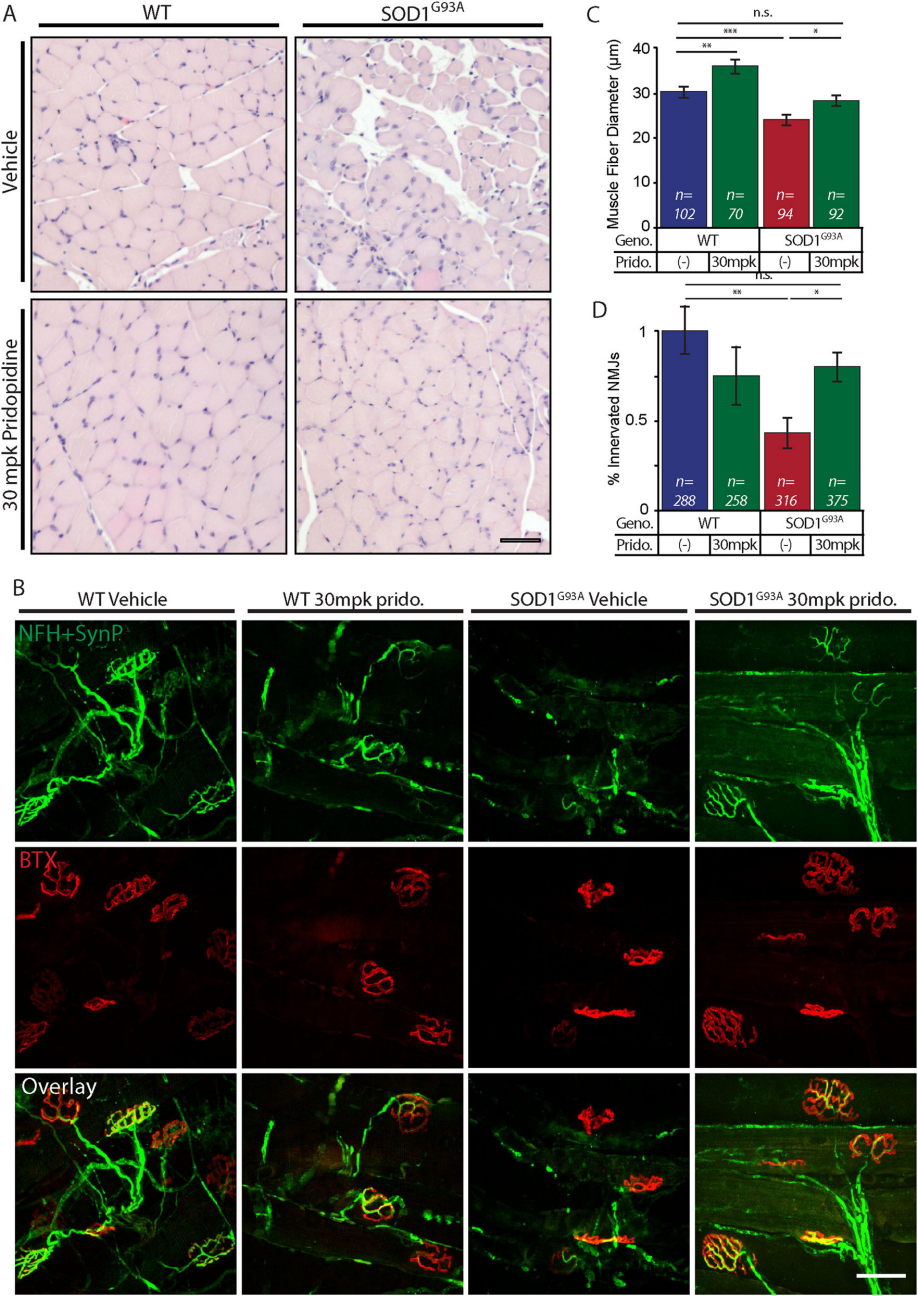
Pridopidine ameliorates muscle fiber wasting and preserves NMJs in 16-week-old SOD1^G93A^ mice. **a** Representative images of H&E-stained cross-sections from Gastrocnemius muscle of mice from 4 groups: WT-vehicle, WT-30mpk pridopidine, SOD1^G93A^-vehicle, and SOD1^G93A^-30mpk pridopidine. The muscle histology of SOD1^G93A^-vehicle mice is poor and reveals a smaller diameter of muscle fiber, compared with WT-vehicle and WT-30mpk pridopidine. Muscles from pridopidine-treated SOD1^G93A^ mice exhibit a larger and more homogenous muscle fiber population that resembles WT muscles. **b** Representative confocal images of muscle whole-mount preparations from 16-week-old mice stained for Postsynaptic AchR (BTX; red) and Presynaptic NFH + Synapsin-I + Synaptophysin (green). Muscles of SOD1^G93A^ vehicle-treated mice show the degeneration of presynaptic neurons and the amorphous post-synaptic apparatus. SOD1^G93A^-30mpk pridopidine-treated mice display co-localized pre- and post-synaptic markers with a typical healthy NMJ morphology. **c** Quantitative analysis of muscle fiber diameter indicates that SOD1^G93A^-vehicle-treated muscle fibers are significantly smaller in diameter than are WT-vehicle fibers. 30mpk of pridopidine led to a significant ~4 µm increase in the muscle fiber diameter in SOD1^G93A^ and ~5 µm in WT muscles. (n = number of muscle fibers.) **d** Quantitative analysis of the percentage of innervated NMJs reveals a massive ~60% loss of NMJs in the SOD1^G93A^-vehicle group, compared with the WT-vehicle group. Pridopidine treatment limited the loss of NMJs in SOD1^G93A^ mice to ~20%. Data are shown as mean ± SEM (*n* = number of NMJs). *p value < 0.05; **p value < 0.01; ***p value < 0.001 (*n* = 5 mice in each group; Student’s *t* test)

NMJ disruption and the subsequent skeletal muscle wasting are key pathologies of ALS. Following our results in vitro, we sought to determine whether NMJs and muscle wasting are also spared in vivo. Gastrocnemius muscles from vehicle or pridopidine-treated (SC) mice were extracted at the age of 16-weeks. Muscle cross-sections were stained with Hematoxylin & Eosin (H&E), and the mean-muscle fiber diameter was quantified for each group. In accordance with previous reports, the muscle fiber diameter of vehicle-treated SOD1^G93A^ mice was markedly smaller than that of WT controls (Fig. 7a–c). Notably, daily SC administration of pridopidine at 30mpk significantly ameliorated muscle fiber deterioration in SOD1^G93A^ mice. Next, we evaluated the number of innervated NMJs in gastrocnemius muscles by co-localizing pre- and post-synaptic markers. Here too, our observations recapitulate previous key reports and indicate significant NMJ disruption, and morphological changes in the post-synaptic apparatus in SOD1^G93A^ muscles (Fig. 7b–d). Strikingly, muscles from pridopidine-treated subjects consist of ~50% more innervated-NMJs, and maintain the healthy morphological traits of the post-synaptic apparatus.

Taken together, our results provide primary evidence for the beneficial outcomes of administering pridopidine to ALS subjects.

## Discussion

**Fig. 8:**
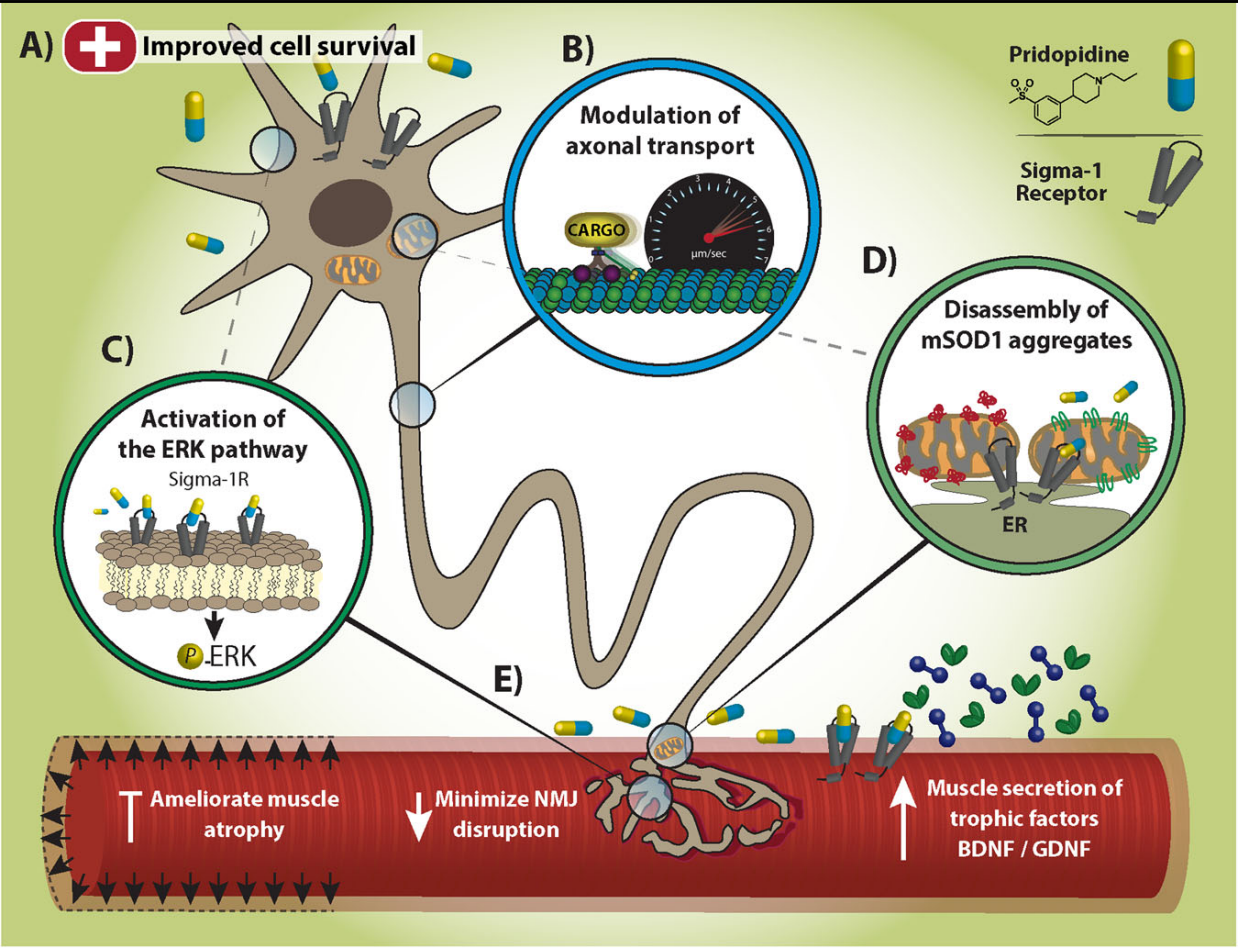
Model. **a** Pridopidine triggers Neuroprotective pathways and promotes survival of SOD1^G93A^ MNs. **b** S1R targeting by pridopidine modulates axonal transport of BDNF, GDNF and mitochondria in SOD1^G93A^ MNs. **c** Pridopidine activates ERK signaling in MNs via S1R. **d** Pridopidine chaperones mSOD1 aggregates. **e** Pridopidine minimizes NMJ disruption and the subsequent muscle atrophy in SOD1^G93A^ models. Pridopidine also increases neurotrophic factor release from muscles

In this study, we identify the potency, and the partial mechanism by which pridopidine conveys its effects in ALS, including NMJ degeneration, MN loss and AT (Fig. 8). Our observations indicate that pridopidine activates the ERK signaling pathway and provides a deeper mechanistic view of previous research that demonstrated the beneficial effects of a S1R agonist in SOD1^G93A^ mice^33^.

### Preservation of NMJs and MN survival

NMJ disruption and distal axonopathy are part of the ‘Dying-back’ mechanism in ALS, which supports the notion that NMJs are the first compartment to fall and to initiate axonal degeneration, followed by neuronal death and muscle atrophy^1^.

Utilizing our unique co-culture method^24,34^, we detect neuromuscular abnormalities in cultures of MNs and myocytes expressing ALS-causative mutations. Remarkably, pridopidine was able to prevent NMJ loss in this model via a S1R-dependent mechanism. Interestingly, high concentrations of pridopidine were able to moderately increase the innervation rate even in co-cultures with S1R^−^^/^^−^ neurons. This observation suggest that at high concentrations pridopidine may bind and act on targets with lower affinity, such as D2R^9^. Alternatively, pridopidine could bind targets in skeletal muscles and promote the release of neurotrophins, Indeed, recent reports reveal that activation of S1R increases the secretion of BDNF and GDNF in various cell types^11,35,36^. Noteworthy, our results indicate pridopidine mildly increases BDNF secretion in the SOD1^G93A^ which may account for the above discussed effects in the co-cultures with S1R^−^^/^^−^ MNs.

One of our striking findings is that the death of MNs occurs in a dying-back mechanism emphasizing the importance of focusing therapeutic attempts on limiting the disruption of the NMJs as a critical break to the consequent MN death. Nevertheless, pridopidine could potentially enhance neuronal survival via glial cells^37^, and therefore, we evaluated MN survival in SOD1^G93A^ co-cultures by exposing all compartments to pridopidine. Our results indicate that a low dose of pridopidine profoundly impedes the observed death of SOD1^G93A^ MNs.

### Axonal transport

Consistent with previous reports of AT deficits in ALS, we demonstrate alterations of AT in SOD1^G93A^ MNs that precede its stereotypic symptoms^26,38^. Importantly, pridopidine was able to enhance AT in both WT and SOD1^G93A^ neurons, and thus improve these deficits. Since we could not detect differences in S1R levels between WT and SOD1^G93A^ MNs, the conflicting results between these two at high (1 µM) concentration of pridopidine could again relate to its binding to low-affinity targets which may be expressed differently between these two in MNs. Therefore, we continued our experiments only with the low dosage of pridopidine (100 nM).

Our data reveal that pridopidine phosphorylates ERK in MNs, similarly to its phosphorylation by neurotrophins^29^ or upon nerve injury^39^. This was previously shown to activate retrograde AT by recruiting Dynein motor-proteins^39^. Optionally, S1R may regulate of Tau phosphorylation by negatively regulating P35 and CDK5^40^, therefore, targeting S1R may reduce tau phosphorylation and generate stable microtubule networks as fast routes for long-distance AT^41^.

According to our data, the retrograde transport velocities of BDNF, GDNF and mitochondria were increased in SOD1^G93A^ co-cultures. Remarkably, pridopidine could modulate the AT properties of these entities, even when applied exclusively on axons/NMJs.

Unlike the immature MN cultures we initially tested, in co-cultures, MNs become active and are also exposed to SOD1^G93A^ muscles; therefor, better mimicking mature motor units. Thus the enhanced retrograde transport of neurotrophins could be due to an active stress mechanism^42^ in SOD1^G93A^ MNs in response to ER-stress^43^, calcium overload^44^ and ROS^45^, which all might be diminished in by pridopidine via its action on the S1R^14^. Another option could be the binding of BDNF to receptors such as p75^NTR^ which may traffic at different velocities, and affect neuronal survival by conveying retrograde death signals^46,47^.

Furthermore, pridopidine strongly repressed GDNF transport in SOD1^G93A^ co-cultures. The *V*_*avg*_ distribution plots (Fig. 4h) indicate the co-occurrence of two transporting populations. Previous reports suggest GDNF plays a dual-spatial role in its action by locally enhancing axonal arborization^48,49^ and innervation rate of muscles, and by distally activating AKT signaling in the cell body through its transport^24,50^. We hypothesize that the reduced stress levels and activation of ERK signaling in SOD1^G93A^ co-cultures treated with pridopidine initiate local mechanisms such as synaptogenesis that depend on local GDNF. The population of fast-moving GDNF may induce survival via AKT activation, and is common for both WT and SOD1^G93A^ treated cultures.

Lastly, enhanced mitochondrial transport could originate in a specific population, which transports faster towards the cell body. This population might consist of damaged mitochondria that need to undergo reparation or degradation^51,52^. Importantly, Bernard-Marissal and colleagues have demonstrated that dysfunction of S1R perturbs AT of mitochondria in MNs^19^. Consistent with the above, S1R are enriched at the MAM, suggesting they might specially control mitochondrial fate.

### Pridopidine, S1R, and ERK activation

Our results demonstrate that pridopidine activates the ERK pathway in a S1R-dependent manner^11^. However, we could not detect the reported pridopidine-dependent AKT activation. Our recent publication revealed that ERK activation occurs following TrkB phosphorylation at the plasma membrane, whereas AKT activation requires internalization of TrkB into endosomes^53^. Recent reports illustrate that agonizing S1Rs can activate TrkB in both BDNF-dependent^31^ and independent^54^ manners, consequently activating ERK. Testing whether pridopidine functions similarly, we were not able to detect the TrkB activation. Future research will have to address the intriguing question of whether pridopidine activates TrkB and subsequently ERK through an alternative pathway independent of BDNF.

### Aggregates and mitochondrial dysfunction

A key finding in this study is the strong reduction in mSOD1 aggregates in the spinal cords of diseased mice treated with pridopidine. The formation of protein aggregates is a common feature in several genetic mutations that cause familial ALS^4^, and it was also reported to occur in sporadic forms of ALS^55^. Protein aggregates are hallmarks of several other neurodegenerative diseases, e.g., Huntington’s, Parkinson’s, and Alzheimer’s disease^56^. Our results repeatedly imply that pridopidine functions through S1R. Indeed, mutations in S1R cause ALS^21^ and deletion of S1R in mice induces motor deficits, NMJ disruption, defects in AT of mitochondria, axon degeneration, increases α-synuclein aggregation and loss of dopaminergic neurons^13,19^. Strikingly, deletion of S1R in SOD1^G93A^ mice exacerbates the disease’s progression in mice^20^.

This work provides the first evidence for the efficacy of pridopidine in treating ALS via its activity on the S1R (Fig. 8). Although our efforts in testing the survival and weight of orally administered pridopidine in SOD1^G93A^ did not yield any significant effect (Supplementary Figure 4), the promising results we reveal in this work encourage further evaluation of pridopidine in ALS. Additionally, other ALS models, including ALS iPSCs should be tested for assessing the potency of pridopidine in ameliorating disease phenotypes and extending the lifespan.

## Materials and methods

### Animal experiments

HB9::GFP (Stock No: 005029) mice were originally obtained from Jackson Laboratories. The colony was maintained by breeding with ICR mice. SOD1^G93A^ mice (Stock No: 002726) were originally obtained from Jackson Laboratories, and maintained by breeding with C57BL/6 J mice. B6;129S6-*Chat*^*tm2(cre)Lowl*^/J (Stock No: 006410) and B6;129S6-*Gt(ROSA)26Sor*^*tm14(CAG*^^−^^*tdTomato)Hze*^/J (Stock No: 007908) mice were originally obtained from Jackson Laboratories, and were cross bred in the Tel-Aviv SPF animal unit to yield the homozygous ChAT::Rosa^tdTomato^ mice. The ChAT::Rosa^tdTomato^ colony was maintained by in-breeding males and females from the colony. SOD1^G93A-ChAT::tdTomato^ and WT ^ChAT::tdTomato^ embryos were obtained by mating ChAT::ROSA^tdTomato^ females with SOD1^G93A^ males. Embryos were genotyped to distinguish WT from SOD1^G93A^ embryos. Pregnant mSigmaR1 knockout female mice and adult male and females (Stock No: GTA000234) were obtained from Charles River Laboratories. The colony was maintained by breeding males and females within the colony. Mice and embryos were genotyped using the polymerase chain reaction (KAPA Bio systems); DNA samples were generated from ear, tail, or embryonic tissue biopsies.

Animal experiments were performed under the supervision and approval of the Tel-Aviv University Committee for Animal Ethics. The in vivo experiments were conducted under the animal housing and care conditions of Teva Pharmaceuticals: SOP 33.22.01 and 33.22.02 and were approved and carried out in accordance with Israel animal welfare laws by the ethics committee (#684).

### Drugs

Pridopidine HCL (TV-7820, ACR16, Batch #160IN0815A) was dissolved in sterile ddH_2_O. Sterile ddH_2_O was used as a vehicle control.

Riluzole (Sigma; R116; Lot # 057K3900V) was dissolved in sterile DMSO.

### Microfluidic chamber preparation

Polydimethylsiloxane (PDMS) microfluidic chambers (MFCs) for co-culture and AT assays were designed and casted as previously described^24,25,57^.

Briefly, three 7-mm wells were punched, and three small ‘caves’ were further carved in the explant compartment to enable spinal cord explants to anchor and enter the growth channels. MFCs are cleaned first with adhesive tape to remove gross dirt and then soaked in 70% EtOH for 15 min, after which MFCs were dried and UV radiated for 10 min. MFCs were then attached to a 50 mm Fluorodish glass bottom dish (WPI) or to a 60-mm plastic bottom dish. AT assays were performed on a glass bottom dish. All compartments were coated with 1.5 ng/mL polyornithine (P-8638, Sigma) in PBS and left overnight, and then replaced with 150 µL laminin (L-2020, Sigma), 1:333 in deionized distilled water (DDW) overnight. Muscle-neuron co-cultures were performed in a plastic bottom dish.

Muscle channels were coated with Matrigel diluted 1:10 with DMEM containing 2.5% PSN for 30 min at 37 °C, before filling the muscle wells with 150 µL of Bioamf-2 medium (Biological Industries; 01-194-1 A). The explant well and channel were filled with 150 µL of 1.5 ng/mL polyornithine (P-8638, Sigma) in PBS and left overnight, and then replaced with 150 µL laminin (L-2020, Sigma), 1:333 in deionized distilled water (DDW) overnight. One day before plating the spinal cord explant, laminin was replaced with explant medium containing Neurobasal (Life Technologies; 21103049) supplemented with 2% B27 (Invitrogen; 1750404), 1% penicillin-streptomycin (Biological Industries), 1% Glutamax (Life Technologies), and 25 ng/mL brain-derived neurotrophic factor (Alomone Labs), until the day on which co-culturing began.

### Fluorescence microscopy and image analysis

All confocal images were captured using a Nikon Ti microscope equipped with a Yokogawa CSU X-1 spinning disc and an Andor iXon897 EMCCD camera controlled by Andor IQ3 software. Bright field movies of muscle contraction were acquired using the same microscope in wide field mode and images were captured with an Andor Neo sCMOS camera. All live-imaging assays were performed at 37 °C and in a 5% CO_2_ controlled and humidified environment.

### Axonal transport of quantum-dot:BDNF

Axonal transport in ventral spinal cord explants was tracked using a modified version of a previously described procedure in dorsal root ganglia^57,58^. Briefly, ventral spinal cord explants from mouse embryos at embryonic day 12.5 (E12.5) were cultured and plated in the MFCs. At 4 DIV the medium in both compartments of the MFC was refreshed and either pridopidine (0.1 µM / 1 µM; Teva Pharmaceuticals) or Riluzole (25 µM / 100 µM; Sigma, R116) was added to all compartments for overnight incubation. Medium in control chambers was refreshed and the chambers remained untreated. The next day, the medium in all wells was replaced with starvation medium (PNB; 1% Glutamax, 1% Penicillin-Streptomycin in Neurobasal) for 1.5 h. Fresh Quantum-dot:BDNF (Qdot-BDNF) was prepared by mixing Qdot (1 µM; Life technologies, Q10101MP) with Biotin-BDNF (30 ng/µL; Alamone Labs, B-250-B) in a 1:3 ratio. The mixture was incubated for 30 min on ice, then diluted 1:55 with culture medium and applied to the distal compartment of the microfluidic chamber. The volume in the proximal well was kept higher than in the distal compartment to prevent the free-flow of Qdot-BDNF from one compartment to another. Following a 50-min incubation, high-magnification movies of Qdot-BDNF axonal transport were acquired for 5 min each, at a frame rate of 20 frames per min, using a 60X oil objective.

### Axonal transport of BDNF-mCherry, GDNF-GFP and MitoTracker in co-culture

BDNF-mCherry and GDNF-GFP expression vectors were sub-cloned by KeyClone, Ltd. Primary myocytes at 5 DIV were transfected with both BDNF-mCherry and GDNF-GFP overnight using Lipofectamin 2000 (Invitrogen). The next day, muscles were trypsinized and plated in the distal compartment of MFCs and underwent differentiation for another 7 days. On the 7^th^ day, ventral spinal cord explants from WT or SOD1^G93A^ embryos were plated in the proximal compartment of the microfluidic chambers. The NMJ compartment was exclusively treated every other day with either 0.1 µM Pridopidine in PNB medium or PNB medium as a control. One week after co-culturing, 200 nM MitoTracker Deep-Red FM (Molecular Probes; M22426) was added to both compartments for 30 min and then washed away. High magnification, multi-channeled movies of BDNF-mCherry, GDNF-GFP, and MitoTracker axonal transport were acquired for 5 min each, at a frame rate of 20 frames per minute, using a 60X oil objective.

### Axonal transport analysis

Qdot-BDNF particle tracking was performed on Bitplane Imaris, using the semi-automated spot tracking function. Inclusion criteria for particle analysis: Track duration > 10 frames; average velocity ≥ 0.2 µm/s; stop duration: Speed < 0.1 µm/s for 3 frames. Data were then exported to MATLAB for further analysis of particle transport: Instantaneous Velocity, Average Velocity, Stops Count, Run Length, and Mean Square Displacement (MSD).

### Muscle-MN co-culture

Primary muscle culture and co-culturing procedures were performed as previously described^25^. Briefly, SOD1^G93A^ adult female mice, or their healthy littermates were euthanized at the age of 60 days (P60). Gastrocnemius muscles were excised and treated with 2 mg/mL Collagenase-I (Sigma) for 3 h. Muscles were then dissociated into single fibers, and were collected into pre-coated matrigel (Thermo) dishes for a 3-day incubation in a Bioamf-2 + 1% Penicillin-Streptomycin-Nystatin medium. On the 4th, 5th, and 6th days, cultures underwent pre-plating for isolation of pure, fibroblast, and debris-free, myoblast culture. Next, 100,000 myoblasts were plated in the distal compartment of a pre-coated MFC. Cultures were grown and allowed to differentiate for another 7 days prior to the addition of a spinal cord explant to the proximal compartment. Bioamf medium was refreshed every second day. At 13 DIV, ventral spinal cord explants were dissected from E12.5 embryos and plated in the proximal channel of the muscle-containing MFC. On the same day, Bioamf-2 medium for muscles and SCEx for spinal cord explants were added with Pridopidine (either 0.1 µM or 1 µM). Control (“0 µM”) chambers remained untreated. Medium containing Pridopidine was refreshed at 15 DIV. At 17DIV, once axons had already extended into the muscle compartment, medium in both muscle and neuronal compartments was switched to starvation media, with or without Pridopidine at either of the concentrations. Medium was refreshed at 19DIV. At 21DIV, movies of muscle contraction were acquired at a frame rate of 30 frames per second for 1000 frames.

### Muscle contraction analysis

Muscle contractile behavior was analyzed as previously described^25^. Briefly, we examined the contractile activity of muscles in the distal compartment of the MFC; the muscles overlapped by at least one axon. Muscles were categorized into two groups: ‘contracting’ or ‘non-contracting’, depending on their motile activity during the movie. The motility of muscles was validated by generating intensity-over-time plots for each muscle (Fig. 2d). The number of contracting muscle fibers per chamber was divided by the total number of muscle fibers analyzed in the same chamber, yielding the percentage of contracting myotubes as an output of NMJ activity.

### In vitro NMJ quantification

NMJ disruption was determined by imaging the NMJ compartment in co-cultures of WT or SOD1^G93A^ myocytes with ventral spinal cord explants from WT and SOD1^G93A^ embryos expressing tdTomato in ChAT-positive cells (ChAT::Rosa/tdTomato), respectively. Co-cultures were treated every other day either with 0.1 µM Pridopidine in PNB medium or with PNB medium as a control. At 7 days of co-culture, cultures were fixed and the myocytes in the NMJ compartment were stained with α-Bungarotoxin-FITC 1:30 (Alamone Labs; B-100-F) to label the extracellular domain of postsynaptic nAchR. Confocal high-magnification images of myocytes and axons in the distal compartment were acquired, and co-localized ChAT::tdTomato axons with α-Bungarotoxin patches were analyzed. The percentage of healthy NMJs was determined by dividing the number of intact co-localization events, with intact non-degenerated axons, by the total number of co-localization events identified in every MFC.

### Analysis of motor neuron survival

The percentage of surviving motor neurons in co-culture was calculated by counting the ChAT::tdTomato-positive motor neurons within a spinal cord explant over time in co-cultures of WT or SOD1^G93A^ myocytes with WT or SOD1^G93A^ spinal cord explants expressing ChAT::tdTomato, respectively. Briefly, co-cultures were treated every other day with either 0.1 µM Pridopidine in PNB medium or with PNB medium in controls. Confocal multi-stacked images of the same spinal cord explant were acquired at different time points starting at day 7 of co-culture and onwards. The percentage of surviving motor neurons at each time point was calculated by dividing the number of motor neurons in every explant by the number of motor neurons that was initially detected in it.

### BDNF-mCherry secretion assay

Primary myocytes at day 5 in vitro were transfected with BDNF-mCherry as previously described above. Myocyte cultures were then grown for another 7 days. Bioamf 2.0 was used as the growth medium until 9DIV and was replaced with muscle differentiation medium containing 2% Horse Serum, 1% PSN, and 1% Glutamax in DMEM. At 12DIV, cultures were incubated for 16 h with 0.1 µM Pridopidine in muscle differentiation medium (collection medium) at a minimal volume. Media in control cultures was refreshed as well. At the end of incubation, conditioned media was collected and treated immediately with protease inhibitors. Media was then centrifuged once at 400 × g to remove dead cells, and then again at 10,000 × *g* to remove cell debris. The corresponding myocyte cultures for each condition were immediately cooled down and lysed using radioimmunoprecipitation assay buffer (RIPA) containing protease and phosphatase inhibitors. The levels of BDNF-mCherry in the conditioned media and the culture extract were determined by SDS-PAGE.

### Immunohistochemistry

In vitro neuromuscular junctions were stained as previously described^25^. Briefly, co-cultures we fixed with 4% PFA for 15 min. TMR-α-Bungarotoxin 1:100 (Sigma; T0195) was used to mark post-synaptic AChR prior to permeabilization. Permabilization was performed by using 0.5% Triton X100 in PBS for 10 min. Cultures were blocked for 1 h in blocking solution containing 1 mg/mL BSA (Amresco; 0332-TAM), 10% Goat serum, and 0.1% Triton in PBS. Primary antibody labeling was performed over night at 4 °C in blocking solution. Primary antibodies were Chicken-anti-NFH 1:500 (abcam; ab72996) and Rabbit-anti-Ryanodine Receptor 1:400 (Milipore; AB9078). Secondary antibodies used: AlexaFluor 405-anti-Chicken 1:500 (abcam; ab175675). AlexaFluor 488-anti-mouse 1:500 (Jackson; 715-545-151). AlexaFluor 488-anti-Rabbit 1:500 (Invitrogen; A11034)Nuclei were stained with DAPI 1:10,000 (Sigma, D8417). Samples were mounted using ProLong Gold antifade reagent (molecular probes; P36934).

Spinal cord sections were prepared by fixation of adult spinal cords in 4% PFA for 24 h, followed by incubation in 20% Sucrose for 24 h. Spinal cords were embedded in OCT and ~15 µM sections were cryo-sectioned. Lumbar spinal cords sections (L1-L6) were extracted, fixed and embedded for cryosectioning. 10 µm sections were prepared. Sections were permeabilized by 0.5% Triton X100 for 15 min. NSC500 (Psychogenetics^32^) was used to stain the SOD1 aggregates. SOD1 aggregates were analyzed in a blinded fashion.

NMJs in vivo were stained on gastrocnemius muscles as previously described^59^. Briefly, gastrocnemius muscles were fixed for 24 h in 4% PFA. AchR was labeled with TMR-α-Bungarotoxin 594 (1:100; Sigma, T0195) for 15 min. Samples were permeabilized in ice-cold 100% MeOH for 5 min. Blocking was performed by incubating samples in 1 mg/mL BSA (Amresco; 0332-TAM) and 0.1% Triton X100 in PBS for 1 h. Primary antibodies were diluted in blocking solution and incubated with samples over night at 4 °C. Primary antibodies: NFH 1:500 (chicken; Abcam, ab72996); Synaptophysin 1:500 (mouse, Milipore, MAB5258); Synapsin-I 1:500 (mouse, Abcam, MAB1543P). Secondary antibodies used: Alexafluore 488-anti-Mouse (Jackson Laboratories; 715-545-151) Alexafluore 488-anti-Chicken (Abcam; Ab150173). Muscle fibers were spread on a slide and mounted with Vectashield (Vector Laboratories; H-1000). Analysis of the co-localization of NMJs in vivo was performed in a blinded fashion.

### Primary motor neuron culture

Motor neurons were cultured as previously described^59^. Briefly, spinal cord explants from SOD1^G93A^, WT, or Sig1R^−^^/^^−^ E12.5 mouse embryos were collected in 1X HBSS buffer (Gibco), then trypsinized and consequently triturated in L-15 medium (Life Technologies) containing 0.4% BSA (Sigma) and 2–10% DNAse (Sigma). Cells were centrifuged through a 4% BSA cushion and then resuspended in Complete Neurobasal Medium (CNB) containing 2% Horse serum (Biological Industries, Ltd.), 2% B27 Supplement, 1% Glutamax, 1% Penicillin-Streptomycin, 0.5% 2-mercaptoethanol, 1 ng/mL BDNF (Alamone labs, B-250), 1 ng/mL GDNF (Alamone labs; G-240), and 0.25 ng/mL CNTF (Alamone labs; C-240). Motor neurons were isolated by centrifugation through Optiprep gradient (10.4% Optiprep (Sigma-Aldrich), 10 mM Tricine, 4% glucose) for 20 min at 760 × g with the brake turned off. Culture dishes were pre-coated with 1.5 g/mL poly D-L- ornithine (PLO; Sigma-Aldrich) overnight at 37 °C and with 3 g/mL Laminin (Sigma-Aldrich) overnight at 37 °C.

For western blotting, 400,000 cells were plated in two 24-well plate wells per condition. MN cultures at 2DIV were starved for 16 h in PNB medium prior to applying pridopidine or BDNF. At 3DIV, BDNF 50 ng/mL or pridopidine (0.1 µM; 1 µM) was diluted in PNB medium and added to the cultures for 30 min. PNB medium was used as a control.

### Western blotting

Primary motor neuron cultures were lysed with RIPA buffer containing 1x protease inhibitors (Roche, 11697498001) and 1x phosphatase inhibitors (Roche, 04906837001), followed by centrifugation and collection of the supernatant. Protein samples were denatured by boiling in SDS sample buffer, then electrophoresed in SDS-PAGE gels, and finally transferred to a nitrocellulose membrane. Membranes were blocked with 5% (w/v) skim milk in TBS-T for 1 h and then immunoblotted with primary antibodies in 5% skim milk: pERK 1:5 000 (Sigma-Aldrich; M8159l); MAP Kinase (ERK1/2) 1:10 000 (Sigma-Aldrich; M5679); pan-AKT 1:1 000 (CST; #4691); phospho(S473)AKT1 1:500 (abcam; ab81823); phospho(Tyr490/516)Trk 1:500; #4619, Cell Signaling Technology); TrkB 1:500 (BD transduction laboratories; #610101); Sigma receptor B-5 1:300 (Santa-Cruz; #137075); mCherry 1:1 000 (abcam, ab167453), followed by species-specific HRP-conjugated secondary antibodies 1:10 000 (Rabbit 111-035-003; Mouse 715-035-151; Jackson Laboratories) and visualized using myECL Imager (Thermo).

### Histology tissue collection and fixation

Gastrocnemius muscles of 20 samples were harvested and fixed in 4% PFA. The samples were then outsourced for histological morphometric analysis at PathoLogica (Nes Ziona, Israel). All tissues were trimmed into block cassettes and sent to CDX-Diagnostics for slide preparation.

### Slide preparation and histological evaluation

Tissues were trimmed, embedded in paraffin, sectioned at no more than 5 microns thickness, and stained with Hematoxylin & Eosin (H&E). The histological processing was performed by CDX-Diagnostics, Jerusalem, Israel. Image analysis was performed using “Image Pro Plus” Version 6.1, Media Cybernetics, US. Two histologic pictures were acquired from each slide of a cross section of the gastrocnemius muscle, and sent for digital analysis using a 10X magnification. A blinded third party performed the analysis.

### In vivo evaluation of survival and body-mass of mice orally administered (P.O.) with pridopidine

The in vivo evaluation studies were performed at Melior-Discovery LTD.

Evaluation was performed on two cohorts of male mice between week 5 to week 18. Each cohort consisted of 4 groups of 12 mice: 36 hSOD1^G93A^/B6SJL and 12 nTG/B6SJL obtained from Jackson laboratories (hSOD1^G93A^/B6SJL stock #002726). hSOD1^G93A^/B6SJL mice were split to 3 groups of 12 mice each and were treated daily *per os* with either: vehicle, 0.3 or 30 mg/kg of pridopidine. 12 nTG/B6SJL in control group were fed daily with vehicle. Cohorts 1 and 2 were evaluated identically. Body weight of individuals was measured weekly. Survival age was calculated as the age at which they either lost righting (in which case the animal was euthanized that day) or were found dead. Those mice were considered “failures” for the survival analysis, while animals that survived to the end of the study and were taken down were considered “censored”. Since none of the WT-Vehicle mice lost righting or died before takedown, the age at takedown for this group is shown for reference.

## Supplementary information

Supplementary Figure 1

Supplementary Figure 2

Supplementary Figure 3

Supplementary Figure 4

Supplemental figure legends
