# Supplementary figures and images for: Targeting the Sigma-1 Receptor via Pridopidine Ameliorates Central Features of ALS Pathology in a SOD1G93A Model
Source: Cell Death Dis. 2019 Mar 1;10(3):210. doi: 10.1038/s41419-019-1451-2 (PMC6397200; doi:10.1038/s41419-019-1451-2)

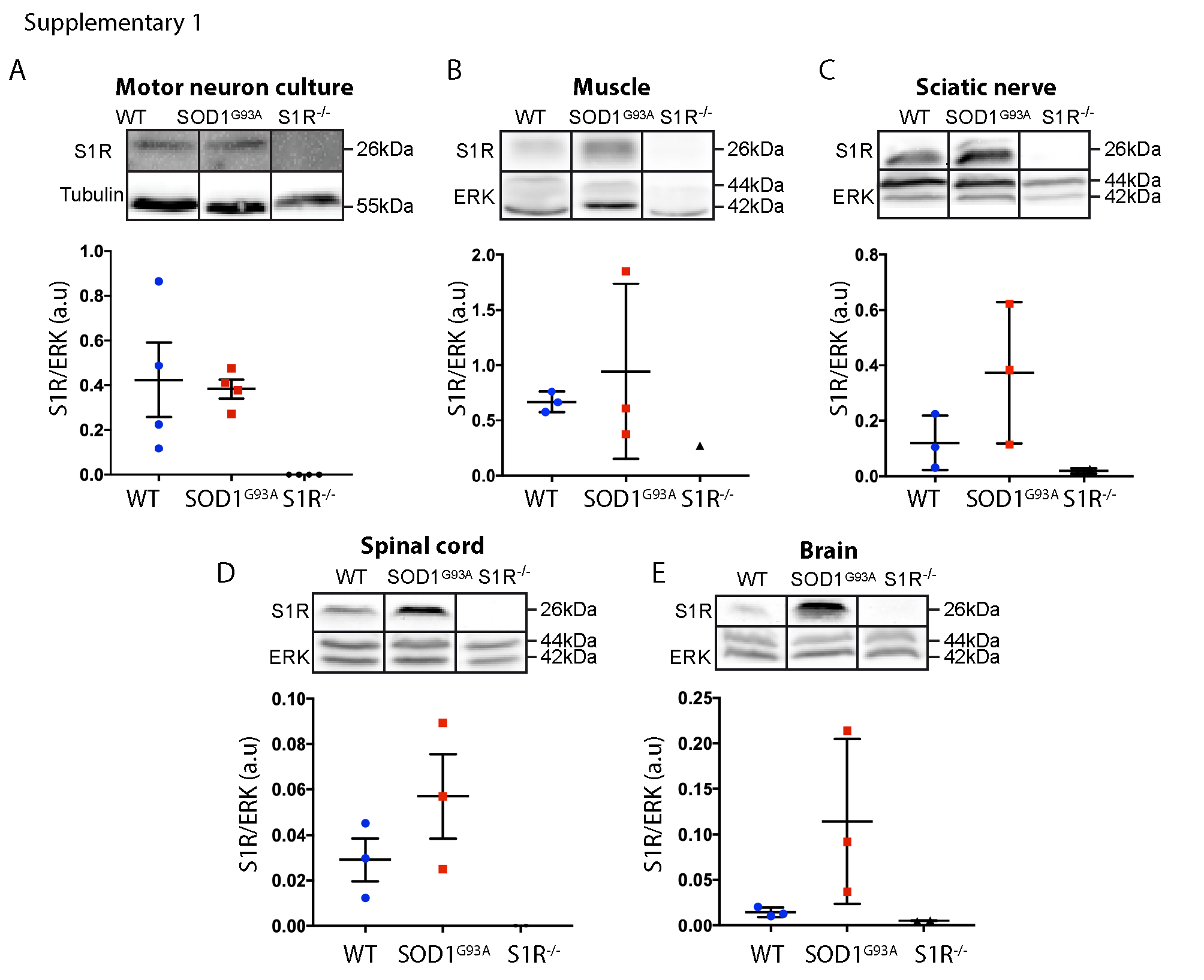

Supplement: Supplementary file 1 — Supplementary Figure 1 [file 41419_2019_1451_MOESM1_ESM.tif]

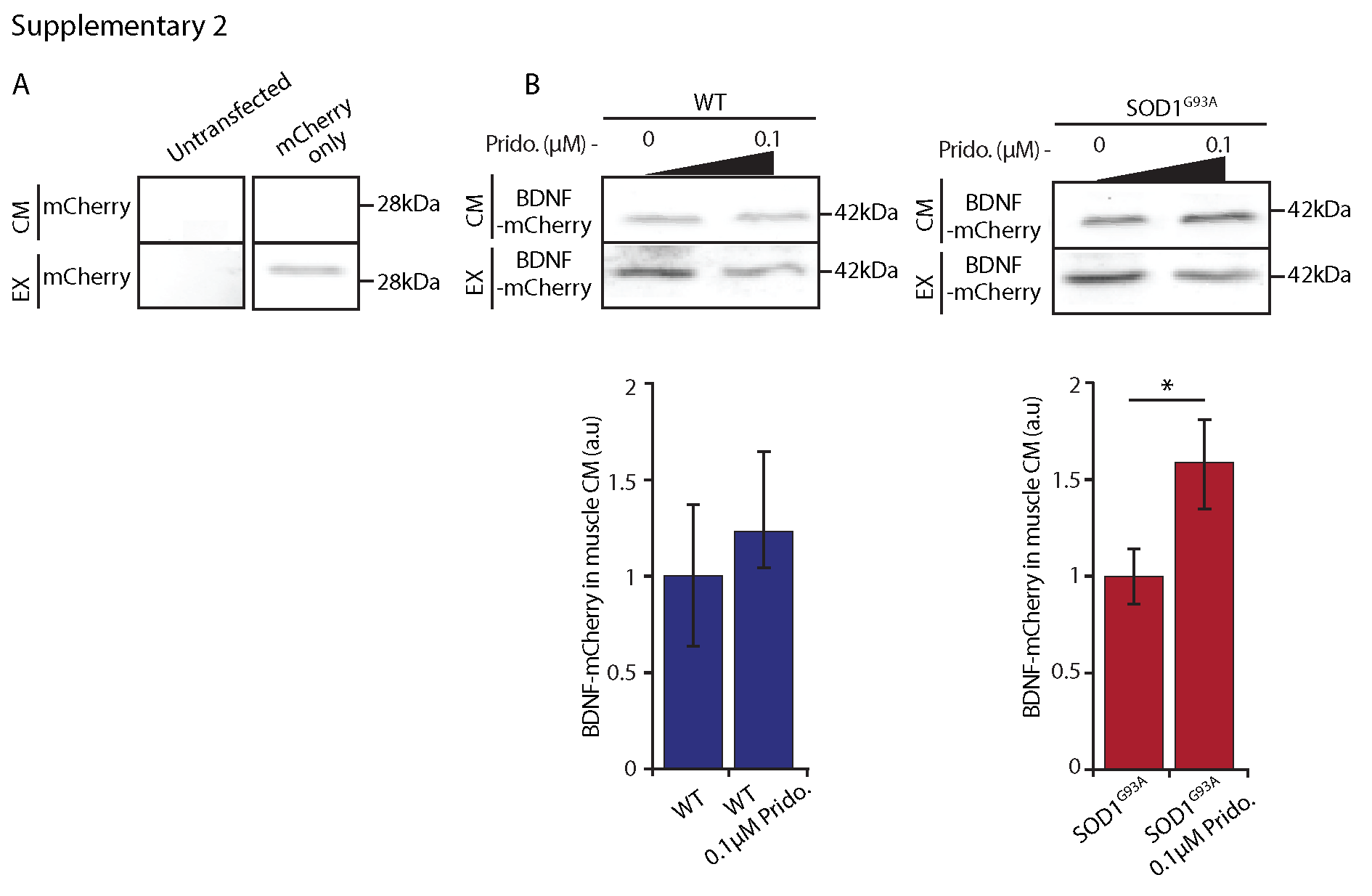

Supplement: Supplementary file 2 — Supplementary Figure 2 [file 41419_2019_1451_MOESM2_ESM.tif]

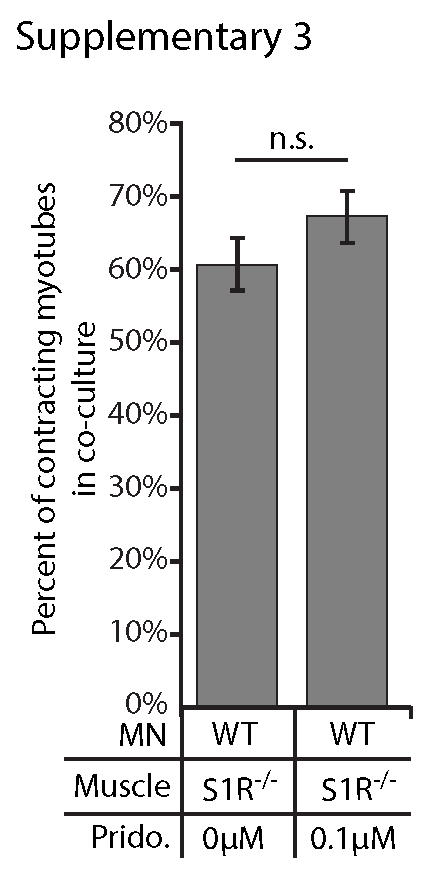

Supplement: Supplementary file 3 — Supplementary Figure 3 [file 41419_2019_1451_MOESM3_ESM.tif]

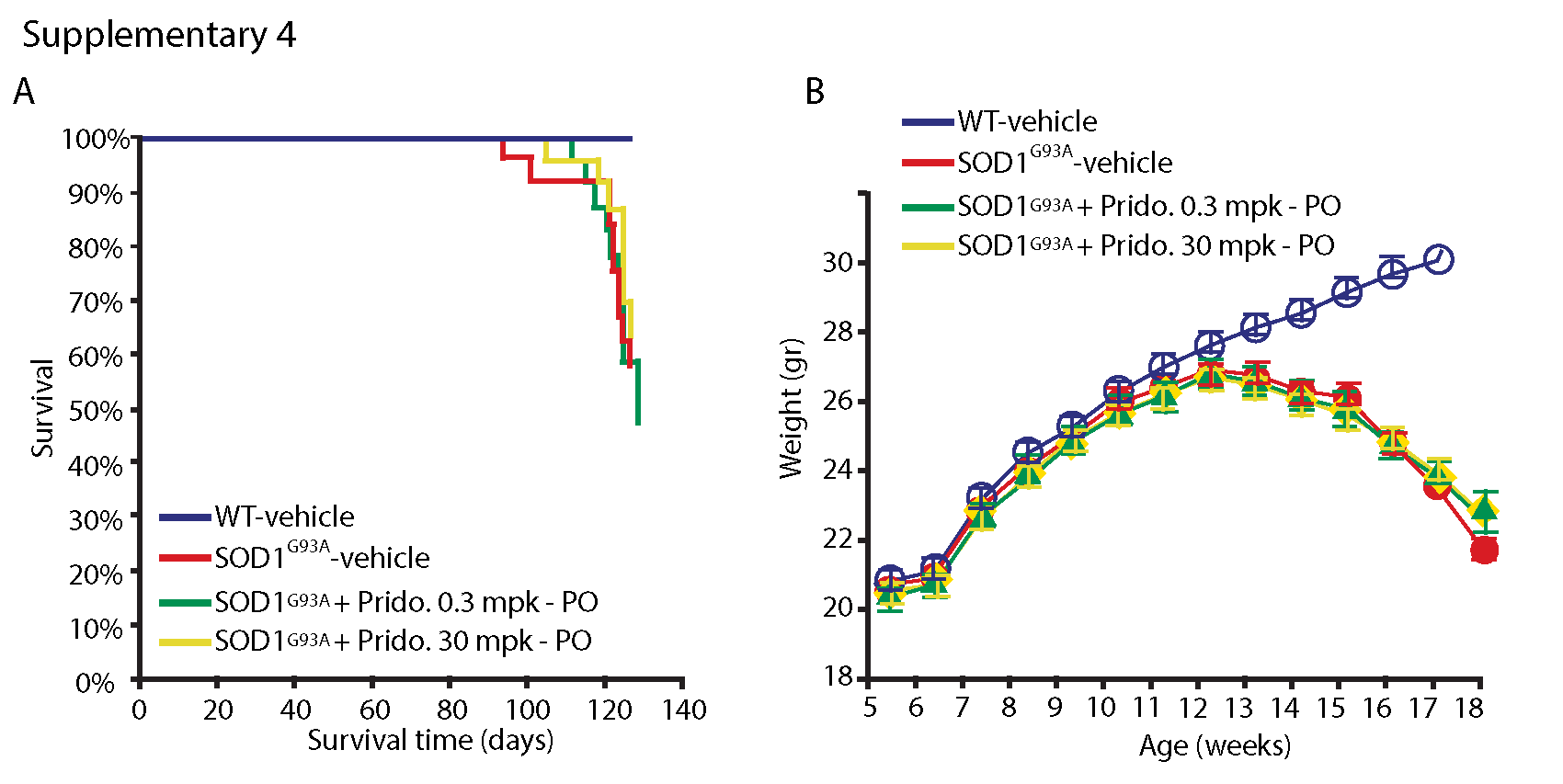

Supplement: Supplementary file 4 — Supplementary Figure 4 [file 41419_2019_1451_MOESM4_ESM.tif]
