# Supplementary material for: Targeting the Sigma-1 Receptor via Pridopidine Ameliorates Central Features of ALS Pathology in a SOD1G93A Model
Source: Cell Death Dis. 2019 Mar 1;10(3):210. doi: 10.1038/s41419-019-1451-2 (PMC6397200; doi:10.1038/s41419-019-1451-2)
Supplement: Supplementary file 5 — Supplemental figure legends [file 41419_2019_1451_MOESM5_ESM.docx]

**Supplementary Figure 1 – S1R protein levels are not altered in SOD1^G93A^ motor neurons.** A) Western blots of WT, SOD1^G93A^, and S1R^-/-^ MN culture extracts show no difference in the S1R protein levels.

B-E) Western blots of (B) gastrocnemius muscles, (C) sciatic nerves, (D) spinal cords, and (E) brains from WT, SOD1^G93A^, and S1R^-/-^ adult mice at P60 (pre-symptomatic SOD1^G93A^) reveal high variability and no significant difference in the levels of S1R protein in SOD1^G93A^ tissues. Lower panel under each blot shows scatter plots for each sample. Data are shown as mean ± SEM.

**Supplementary Figure 2 – Pridopidine increases the secretion levels of BDNF-mCherry from SOD1^G93A^ cultured myocytes.** A) Western blots of untransfected (left) and mCherry-only transfected (right) primary myocytes and their conditioned media indicate that no mCherry band (28kDa) can be detected in these myocyte-conditioned media, whereas mCherry is detectable in the culture extract of the mCherry-only transfected myocytes and is not in the untransfected myocyte culture extract. B) Western blots of conditioned media and the culture extracts of WT primary myocytes that were transfected with BDNF-mCherry display a clear band of BDNF-mCherry (42kDa) in the myocyte conditioned, as well as in the culture extract. Quantitative analysis of BDNF-mCherry levels in conditioned media reveals no significant increase in its secretion following overnight treatment with pridopidine at 0.1µM concentrations. C) Western blots of conditioned media and culture extracts of SOD1^G93A^ primary myocytes transfected with BDNF-mCherry demonstrate a moderate increase in BDNF-mCherry secretion. Data are shown as mean ± SEM (n=3 independent experiments).

**Supplementary Figure 3 – Pridopidine does not influence the percentage of contracting myocytes in co-culture with S1R^-/-^ myocytes and WT spinal cord explants.** A) Bar chart showing the percentage of contracting myocytes in co-culture with a WT spinal cord explant with primary myocytes from S1R^-/-^ adult mice indicates that 0.1µM pridopidine does not alter the percentage of contracting myocytes in this co-culture. Data are shown as mean ± SEM (n=3 independent experiments)

**Supplementary Figure 4- PO administration of pridopidine to SOD1^G93A^ mice does not improve disease outcome.** A) Kaplan-Meier survival plot showing that pridopidine, when orally administered at 0.3mpk or 30mpk does not prolong survival of SOD1^G93A^ mice. B) Body-weights over-time of SOD1^G93A^ mice orally administered with pridopidine demonstrates that this paradigm does not prevent stereotypic decrease in body-mass.
